# Cerebral Vein Thrombosis Misdiagnosed and Mismanaged

**DOI:** 10.1155/2012/210676

**Published:** 2012-04-05

**Authors:** P. K. Sasidharan

**Affiliations:** Department of Medicine and Haematology, Calicut Medical College, Calicut 673008, Kerala, India

## Abstract

Cerebral venous thrombosis (CVT) should be considered in the differential diagnosis of all unexplained CNS disorders of sudden onset. Etiological factors are often subclinical forms of several common thrombophilic states occurring together, rather than the typical inherited and rare causes. Diagnosis is missed because of the heterogeneity in clinical presentation and etiological factors. In several patients with the so called idiopathic CVT nutritional deficiencies and lifestyle issues are more important factors in pathogenesis, rather than single rarer causes. High index of suspicion is the key to diagnosis. Clinical skill has to be fine tuned to diagnose the problem and to identify all the etiological factors. Radiology is essential for diagnosis but relying on radiology alone will lead to missing several cases and even erroneous diagnosis. It is inappropriate to proceed prematurely to laboratory investigations, forgetting proper clinical evaluation by studying diet, lifestyle, and environment of the patients. Success in managing lies in identifying all the contributory causes and correcting all of them giving excellent outcome almost always. Clinical observations based on case series and sharing of such information alone are the means to arrive at a consensus in diagnosis and management.

## 1. Introduction

Thrombosis of the cranial venus sinuses and the cerebral cortical veins can lead to a distinct cerebrovascular disorder, which unlike arterial stroke, most often affects even young adults and children. Symptoms and clinical course are highly variable, etiological factors are even more heterogeneous making cerebral cortical vein thrombosis (CVT) a unique clinical entity. The disorder can occur de novo as the first manifestation or can overlap on another existing clinical problem. In either case it is always multifactorial and is variable in each patient. Each component of the Virchow's triad (endothelial damage, stasis and hypercoagulability of blood) may in turn have several contributory factors/causes to produce the final manifestation of CVT [1]. These factors, which vary from patient to patient, operate together incidentally or accidentally to produce cerebral cortical vein thrombosis and therefore the patients are often not comparable. This also implies that randomized studies are not reliable in those with CVT to arrive at logical conclusions on therapeutic guidelines and strategies. The set of actions required to correct the contributory factors will then be different in each patient. Clinical observations based on case series and sharing of such information alone are the alternative to arrive at a consensus. Because of the heterogeneity in the clinical presentation and etiology, the diagnosis of CVT is often missed, and even if a diagnosis is made the contributory factors which are often subclinical are also missed or overlooked. Most often only one of the etiological factors is prominent enough to be picked up and it is a universal practice to look for rarer causes and some inherited causes of venous thrombosis. Diagnosis is often missed unless clinicians maintain a high index of suspicion and be aware of the varied clinical presentations to be able to recognize and manage by prompt and proper application of clinical skill, rather than depending heavily on investigations alone for effective management of these patients.

Depending on the site, size, duration, and rapidity of development of thrombus, it can present as seizure alone, space occupying lesion, benign intracranial hypertension, subarachnoid haemorrhage, unexplained altered sensorium, or meningoencephalitis [2]. It can be mistaken for metabolic encephalopathy and vertebrobasilar insufficiency. Moreover, the patient may land up with any practitioner or specialist because it can coexist with several medical, surgical and obstetric conditions like pregnancy, eclampsia, postoperative phase, uncontrolled diabetes, pyogenic or other meningitis, DIC, septicemias, hyperviscosity, and thrombophilic states, collagen vascular disorders, and vasculitic syndromes. It could also occur while on treatment with certain drugs like L Asparaginase and Thalidomide. CVT can be the first manifestation of a thrombophilic state like polycythemia, thrombocytosis (both primary and secondary), leukocytosis, Sickle cell disease, hyperhomocysteinemia, haemoconcentration due to a multitude of causes, hypereosinophilia, and APLA syndromes [2]. As mentioned already almost always more than one cause coexist in each patient, which are found out only on good clinical evaluation and not by lab studies alone. To compound all this the new generation doctors forget the importance of proper clinical evaluation and correlating important observations in the diet, lifestyle, and environment of the patients. Many of them do not bother to find out such information and rather proceed prematurely to laboratory investigations to arrive at a diagnosis and they expect the radiologist to confirm the diagnosis always and the laboratory alone to finally pin-down the basic etiology of cerebral cortical vein thrombosis. After making a diagnosis of CVT the clinician should apply clinical skill and common sense with which it is possible to arrive at one or more completely correctable common etiological factors contributing to the development of CVT, even if there is an underlying inherited disorder which cannot be corrected, and thus can avoid recurrences in future. Once the diagnosis of CVT is made it is easily managed if we know all the contributory factors and almost always has a good prognosis as compared to other cerebrovascular accidents. It is also an observation that in many patients with the so called idiopathic CVT nutritional deficiencies and lifestyle issues are more important basic etiological factors in pathogenesis, at least in some epidemiological settings as strict vegetarians and those who consume a unbalanced diet. Research by observation and studying the patients for their diet, lifestyle and environment might give the answer to the several etiological factors in cerebral cortical vein thrombosis, as in all other clinical problems, rather than depending on the costly laboratory investigations alone [2].

## 2. Pathogenesis

**Figure 1:**
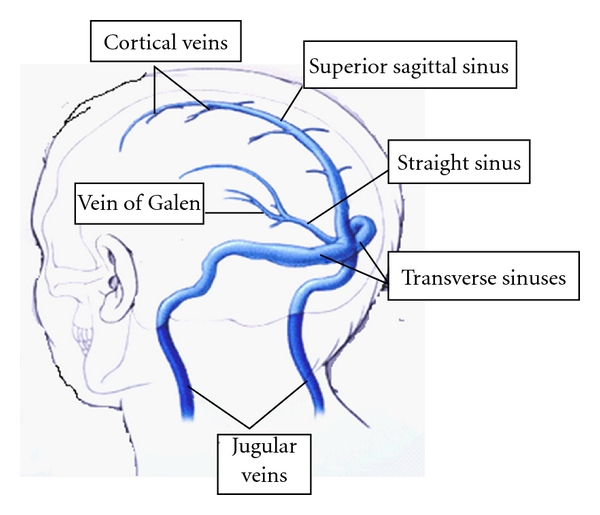
The major venous sinuses and their tributaries.

The dural sinuses that are most frequently thrombosed are the superior sagittal sinus, the lateral sinus (transverse sinus and sigmoid sinus), and cavernous sinus. Less frequently affected are the straight sinus and the vein of Galen. Still rarely smaller cortical veins may be the primary site of thrombus formation without evidence of thrombus in the major sinuses or the thrombus in the major sinus would have resolved by the time the patient comes to clinical attention. This is one reason for the misdiagnosis in CT and MRI. Occlusion of a venous sinus and/or cortical vein is usually caused by a partial thrombus or an extrinsic compression that subsequently progresses to complete occlusion [1]. Once the sinus is occluded, the thrombus may extend to the cerebral cortical veins draining into that sinus (Figure 1). Thrombosis and complete occlusion results in cortical venous infarction, with petechial haemorrhages or overt hemorrhagic infarction [1]. There are different mechanisms for the signs and symptoms in patients presenting with cerebral venous thrombosis: (1) local effects caused by the venous obstruction, consequent ischemia, infarction or hemorrhage, and the neuronal dysfunction; (2) the mass effect behaving like an intracranial space occupying lesion (ICSOL); (3) venous obstruction leading to sudden development of oedema inside the closed compartment of the cranial cavity, which varies depending on the size and site of thrombus, causing rapid development of intracranial hypertension and its consequences; (4) sudden development of oedema and ischemia in the cortex corresponding to the territory involved causing hyper excitability with seizure discharges and later neuronal dysfunction; (5) symptoms and signs due to the underlying disorder/disorders responsible for thrombophilic state. In the majority of patients, all these happen suddenly and simultaneously or sequentially or one of them dominate in the clinical picture. Unlike in arterial obstruction, intracranial hypertension, especially when one of the major venous sinuses is obstructed, is a very common and early manifestation [2]. Cytotoxic edema is caused by ischemia, which damages the energy-dependent cellular membrane pumps, leading to intracellular swelling. Vasogenic edema is caused by a disruption in the blood-brain barrier and leakage of blood plasma into the interstitial space which is reversible if the underlying condition is treated promptly. Besides the severe cerebral oedema of sudden onset, there is another mechanism for the development of intracranial hypertension especially when the thrombus does not resolve or gets organised. Normally, the CSF is transported from the cerebral ventricles through the subarachnoid spaces at the base and surface of the brain to the arachnoid villi, where it is absorbed and drained into the venous sinuses [2]. Thrombosis of these venous sinuses leads to impaired absorption of CSF and, consequently, increased intracranial pressure. Pathological examination shows enlarged, swollen veins, edema, ischemic neuronal damage, and petechial hemorrhages, which can merge and become large hematomas [1, 2].

## 3. Causes and Risk Factors

Why does the thrombus develop? Venous thrombosis anywhere in the body results from exaggerated activity of one or more mechanisms of haemostasis or reduced activity of one or more natural antithrombotic mechanisms or a combination of both [1]. In reality, thrombosis is almost never due to a single etiological factor, it occurs only when several etiological factors come together accidentally or incidentally. In normal state the fluidity of blood is maintained by the following factors: (1) normal intact endothelium; (2) prostacycline; (3) antithrombin III (4) heparan sulfate; (5) thrombomodulin; (6) protein C and Protein S; (7) fibrinolytic System; (8) normal flow of blood; (9) absence of a prethrombotic/hypercoagulable state (thrombophilic state). The common conditions with tendency for thrombosis (thrombophilic states) to be routinely looked for are dehydration (even subclinical), unusual postures in travel or sleep, prolonged immobilisation, surgery or trauma, focus of infection/inflammation/abscess adjacent to the sinuses or in its drainage areas, pregnancy/postpartum period, hyperhomocysteinemia, polycythemias, thrombocytosis, obesity, diabetes, oral contraceptives, hormone replacement therapies, antiphospholipid antibodies, PNH, Behcet's and other vasculitis, congestive cardiac failure, nephrotic syndrome, smoking, Inflammation, liver disease (acquired protein C/S, ATIII def), increasing age, dyslipidemia, atherosclerosis, and malignancies [1, 2]. Rarely only one of the contributory causes is an underlying hereditary thrombophilia- like Factor V Leiden (APCR), prothrombin gene mutation, hereditary hyperhomocysteinemia, deficiency of Protein C, Protein S, antithrombin deficiency, increased factor VIII, and dysfibrinogenemia. But in practice these hereditary thrombophilias are not the primary cause in any patient with thrombosis. One or more of the acquired causes are the real culprit even when they have an underlying hereditary thrombophilia. A clinically obvious prothrombotic risk factor or a direct cause is identified in about 85 percent of patients with cerebral venous thrombosis [1–3]. Even in them there are other several coexisting subclinical factors which need to be corrected to prevent its recurrence. Most often a precipitating factor, such as a head injury or obstetrical delivery, causes cerebral venous thrombosis in a person with another acquired or genetically increased risk. One example is hyperhomocysteinemia which is very common due to poor intake of folic acid containing diet, which is almost universal and B12 deficiency in some strict vegetarians who do not take a balanced vegetarian diet containing green leafy vegetables [4]. When such women become pregnant several other thrombophilic states are added on to the existing ones and therefore deep vein thrombosis or cerebral cortical vein thrombosis occurs. Deficiency of folic acid, B6, and B12 is the commonest cause of hyperhomocysteinemia rather than inherited causes of it [4]. Besides that hyperhomocysteinemia is seen also in many other conditions like, renal failure/liver disease, hypothyroidism, smoking, excessive coffee intake, Inflammatory bowel diseases, psoriasis, and rheumatoid arthritis. Similarly, the commonest cause for Protein C and S deficiency is liver disease which often goes unrecognized, because subclinical liver disease is very common due to alcohol and the Non-alcoholic fatty liver disease. Acquired protein S deficiency can also be due to oral contraceptive pills or hormone replacement therapy, Pregnancy, oral anticoagulants, DIC, nephrotic syndrome, inflammatory conditions, after an acute thromboembolism, autoantibodies to protein S following varicella, and other infections. Antithrombin deficiency when it is severe can have severe arterial and venous thrombosis and resistance to heparin therapy can be there. But after an acute thrombosis, or while on heparin therapy—ATIII levels are <50%, leading to wrong diagnosis. Acquired causes of ATIII deficiency is seen in liver disease, DIC, nephrotic syndrome (loss in urine) chemotherapy with L Asparaginase and preeclampsia. Elevated factor VIII levels can also contribute to thrombosis and is seen in increasing age, obesity, pregnancy, surgery, chronic inflammation, liver disease, hyperthyroidism, diabetes, and in non-O-blood groups [1, 2].

 Pregnancy and postpartum period are important considerations in women of childbearing age. Pregnancy itself is a thrombophilic state due to the changes in blood like Increased factor VIII, fibrinogen, decreased Protein S, decreased fibrinolysis (Increased PAI), obesity, caesarean section, immobilization and other mechanical and haemodynamic factors due to pregnant state [5]. Coexisting hyperhomocysteinemia is very common due to malnutrition and consequent deficiency of folic acid [4]. Besides the chance of tissue thromboplastin entering the blood from placenta due to injury, ischemia, or during delivery of fetus and the coexisting reactive thrombocytosis due to haemorrhage or iron deficiency state-all contribute to increased thrombotic tendency. During the last trimester of pregnancy and after delivery, the risk of cerebral venous sinus thrombosis is increased. The frequency of peripartum and postpartum cerebral venous sinus thrombosis is about 12 cases per 100,000 deliveries [5].

 Inflammatory bowel diseases such as Crohn's disease and ulcerative colitis are described as risk factors. Corticosteroids used in treatment of these conditions may play a causative role. Collagen vascular diseases such as systemic lupus erythematosus, Wegener granulomatosis, and Behcet syndrome have been reported to be associated with CVT [1, 2]. Trauma like head injury, direct injury to the sinuses or the jugular veins (from jugular catheterization), and neurosurgical procedures can be triggering the onset of CVT. Lumbar puncture is postulated to produce CVT sometimes, and some of the postlumbar puncture headaches could be due to CVT [1, 2, 6]. A plausible reason is that low CSF pressure after an LP causes a downward shift of the brain, with traction on the cortical veins and sinuses. Deformation of the venous walls by any mechanism may induce thrombosis. The diagnosis of sinus thrombosis after a lumbar puncture is difficult, because the headache that follows is attributed not to sinus thrombosis but to the lumbar puncture itself. The classical postLP headache typically disappears when the patient lies down, and resolves within a few days. Headache in patients with cerebral venous sinus thrombosis does not change with a shift in posture and it worsens during the early stages and could be more in early mornings. Infections like otitis and mastoiditis can be complicated by thrombosis of the adjacent sigmoid and transverse sinuses [1, 2]. If the contralateral transverse sinus is hypoplastic (a frequent anatomical variant), absorption of CSF becomes impaired and hydrocephalus can result (which is formerly known as otitic hydrocephalus). Spread of infection into the cerebral venous sinuses may occur by extension from the paranasal sinuses. These cases also may be associated with subdural empyema. Bacterial meningitis as a coexistent condition should be considered in these cases. Frontal sinuses are the most common source of infection. A special case is thrombosis of the cavernous sinuses, which is nearly always caused by an infection of the paranasal (ethmoid and sphenoid) sinuses, the orbit, or the face. Multiple organisms are to be considered, staphylococcus aureus being the most common. In chronic infections, Gram-negative organisms and fungi such as Aspergillus species may be found. The frequency of infectious sinus thrombosis has declined in adults. Higher frequencies of both systemic infections (e.g., neonatal sepsis) and local infections (e.g., otitis) are reported in children. Though lateral sinus (transverse and sigmoid) and cavernous sinus thrombosis is usually secondary to infections in the adjacent areas like otitis media, mastoiditis and sinusitis, the superior sagittal sinus thrombosis is most often due to non infective processes. But infection may reach superior sagittal sinus from the nasal sinuses or as extension from the lateral or cavernous sinuses or from epidural or subdural focus of infection. Any of the cerebral venous sinuses or the cerebral veins which drain into the sinuses may be occluded partially or completely by trauma or tumours. The coexisting thrombophilic states like malnutrition or dehydration, hyperhomocysteinemia, and so forth can accelerate the process of thrombosis. The thrombus can then grow into the sinus or vice versa. Transverse sinus and sigmoid sinus thrombosis causes headache and vomiting with or without fever and features of raised intracranial pressure. Papilloedema develops which is usually bilateral (but can be unilateral), drowsiness, coma, and seizures can occur. Cavernous sinus thrombosis, usually originates from infections in the orbit, nasal sinuses, or upper half of the face. The infection commonly involves only one sinus at the onset but rapidly spreads through the circular sinus to the other side. One or both of these can be involved by spread of infection or spread of thrombus from the other dural sinuses. Thus cortical vein thrombosis or any venous or arterial thrombosis is just a manifestation of multiple thrombophilic states which varies from patient to patient and a highly individualised approach is essential for effective management and prevention [1–3, 5–7].

## 4. Clinical Features

Most common venous sinus to develop thrombosis (or probably detected commonly) is the superior sagittal sinus [1, 2, 8]. Irrespective of the sinus affected there are overlapping of clinical features except when it is confined to cavernous sinus or one of the smaller cerebral veins where there will be distinct focal symptoms and signs depending on the area affected. Headache is the most frequent but least specific symptom; severe headache is present in more than 90 percent of adult patients [2, 6]. Usually the headache mimics migraine but is persistently unilateral or diffuse and is not relieved after sleep, increases gradually over a couple of days, but can also start in a split second, mimicking intracerebral or subarachnoid hemorrhage [6]. Nausea and vomiting may be associated. CVT can present as isolated intracranial hypertension syndrome, which is often misdiagnosed as benign intracranial hypertension (headache with or without vomiting, papilledema, and visual problems). Focal syndrome (focal deficits, seizures, or both) mimicking and often mistaken for Intracranial space occupying lesion is not uncommon; even on CT and MRI the venous infarct is mistaken for glioma or even focal demyelination [1, 2, 4, 9]. Encephalopathy with multifocal signs, mental status changes, stupor, or coma can occur suddenly over hours or days. Cerebral lesions and neurologic signs develop in half of them. Unilateral hemispheric symptoms such as hemiparesis or aphasia, followed within days by symptoms due to involvement of the other hemisphere, caused by the development of cortical lesions on both sides of the superior sagittal sinus are characteristic, but rare. Superior sagittal sinus thrombosis may also present as a unilateral lower extremity weakness or paraplegia [1, 2]. Venous infarct has a higher risk of haemorrhagic transformation with subarachnoid leak and may present clinically as a subarachnoid haemorrhage as well [10]. Unlike arterial thrombosis strict localization to one vascular territory may be absent often. Cranial nerve syndromes are seen with lateral venous sinus (sigmoid and transverse sinus) thrombosis. These include—vestibular neuronopathy, pulsatile tinnitus, unilateral deafness, double vision, facial weakness and obscuration of vision. If the thrombosis in lateral sinus extends to the jugular vein, the patient may develop involvement of cranial nerves IX, X, XI, and XII due to Jugular foramen syndrome. If it is secondary to a septic focus there could be fever and chills and other features of septicemia. The classic symptoms of lateral sinus thrombosis are fever, headache, nausea, and vomiting. Seizures occur in about forty percent, which is focal in 50 percent, but may generalise to a life-threatening status epilepticus [9]. Thrombosis of the deep venous system (straight sinus and its branches) causes often bilateral thalamic lesions, with behavioral symptoms such as delirium, amnesia, and mutism, which can be the only manifestation. If large unilateral infarcts or hemorrhages compress the diencephalon and brain stem, patients may become comatose or die from cerebral herniation [1, 2]. Other causes of coma are diffuse involvement and generalized seizures followed by postictal coma. Infectious cavernous sinus thrombosis is characterized by headache, fever, and eye symptoms such as periorbital edema, proptosis, chemosis, and paralysis of eye movements due to involvement of the oculomotor, abducent, or trochlear nerves. Lateral sinus thrombosis may present with increased intracranial pressure as the only presenting sign with associated headache and a pseudotumor cerebri-like picture [1, 2]. Patients with isolated intracranial hypertension have headache but no other neurologic symptoms, with the exception of diplopia due to sixth nerve palsy when the intracranial pressure is very high. Fundus examination may show papilloedema. Severe papilloedema can cause transient visual impairment, or if it is persistent and left untreated can cause even permanent blindness. Besides all these the clinical picture will usually be associated with the underlying disorder/s which contributed to the prothrombotic state, and as mentioned before there will always be multiple factors contributing to that. If the thrombosis occurs in the setting of meningitis, brain abscess, or uncontrolled diabetes the clinical picture will be even more confusing. Extension of the thrombus from the sagittal or lateral sinus into the cerebral veins is almost always accompanied by dramatic signs and symptoms due to hemorrhage into the cortical white and grey matter. Convulsions often are focal, hemiplegia, aphasia, or hemianopia can occur [1, 2].

**Diagnosis**

A high index of suspicion is absolutely essential to diagnose cerebral venous thrombosis. Diagnosis of CVT should be considered in all young and middle-aged patients with recent onset unusual headache, with stroke-like symptoms, especially with seizures, more so when it occurs in the absence of the usual risk factors for arterial thrombosis [1, 2, 5, 6, 9]. Diagnosis is basically clinical, one should be aware of the varied clinical presentations and if the clinical picture, is also consistent with CVT—besides the other usual differential diagnosis—the next step is to get a CT or MRI to look for supportive evidence of CVT and to rule out other mimickers. CT may show multiple lesions, some haemorrhagic others radiolucent which could be bilateral often. After ruling out other causes for the clinical presentation or after getting definite proof for diagnosis then start looking for all the contributory factors of CVT. A common mistake is to depend entirely on the radiologist to give a definite diagnosis and on the laboratory to give an etiological diagnosis always. If the clinical picture is highly suggestive, is consistent with or at least not against the diagnosis of CVT and if there is no other definite etiological diagnosis on CT or MRI the patient should be managed as CVT. This is to avoid delay in initiating the specific treatment directed at the thrombus and its precipitating factors. Clinical presentation is highly variable and it is a notorious mimicker, it may be the presenting clinical problem or may be developing while under treatment or observation for another disorder [1–3, 6, 7]. Usually there is a delay in diagnosis, especially in patients with unexplained intracranial hypertension, or in those with CT evidence of hemorrhagic infarcts, especially if the infarcts are multiple and in those presenting like ICSOL or demyelination on CT. The clinical suspicion of cortical vein thrombosis based on sound clinical reasoning is the most important starting point in the diagnosis and the suspicion of CVT should be indicated in the request form when referring for CT or MRI studies. The most sensitive investigation technique to confirm the diagnosis is MRI in combination with magnetic resonance venography [8]. T_1_, and T_2_-weighted MRI may show a hyper intense signal from the thrombosed sinuses if the clot is recently formed. The combination of an abnormal signal in a venous sinus and a corresponding absence of flow on magnetic resonance venography confirms the diagnosis of thrombosis [8]. But it may not be possible to demonstrate the thrombus in the smaller cortical veins on CT or MRI and the effects of it in the brain may be mistaken for ICSOL or demyelination. Thrombus in the major sinus only is picked up by the imaging studies and we cannot expect it to be present at the site when we order for the imaging, it could be resolving or even completely resolved by the time the imaging is done. CT scanning is a useful technique for the initial evaluation basically to rule out other acute cerebral disorders that mimic CVT and occasionally to show lesions which suggest venous infarcts or hemorrhages. Many times CT scan is reported as normal in patients with CVT. Empty delta sign on CECT—that is, enhancement of the collateral veins in the superior sagittal sinus walls surrounding a nonenhanced thrombus in the sinus is suggestive [2, 8]. Dense triangle sign is formed by fresh coagulated blood in the superior sagittal sinus. HRCT may show the thrombus as a hyperintense signal in a sinus or even in the cortical veins (the “cord sign”). CT venography is a promising new technique for creating images of the cerebral venous system [8]. If the diagnosis is still uncertain after MRI or CT venography, cerebral angiography may be indicated. Angiography provides better details of the cerebral veins and hence is useful in the diagnosis of rare cases of isolated thrombosis of the cortical veins without sinus thrombosis. Angiography also shows dilated and tortuous (“corkscrew”) veins, which are evidence of thrombosis downstream in the sinuses [8]. But depending too much on radiological studies alone for making the diagnosis will result in wrong diagnosis sometimes. The posterior ischemic leukoencephalopathy reported by the radiologists appears to be one such instance because on clinical evaluation these patients have all the features of CVT involving the posterior circulation and they also have the clinical setting to develop venous thrombosis. But if the clinical diagnosis is not asserted by the referring clinician, when there is no radiologic proof of the thrombus in the lateral sinus, it is reported as posterior ischemic leukoencephalopathy. This inference is made by observing the clinical features, the clinical setting, and the course and the predisposing conditions in these patients. It is very likely that we fail to demonstrate the thrombus on investigation and this could be explained by dissolution of the thrombus by natural mechanisms by the time MRI is taken and the evidence of the original insult to brain is still persisting on radiological studies. It could also be that demonstration of thrombus in transverse or sigmoid sinus could be difficult. In the clinical evaluation, what usually helps to diagnose CVT is the dramatic onset to suggest a CVA and the lack of specific localization to a specific arterial territory, clinically or by radiological methods. During evaluation one should take a detailed history to look for any of the clinical settings for an increased risk of venous thrombosis, like history of decreased fluid intake, or increased fluid loss like excessive sweating, diarrhea or vomiting, drug intake including OCP, travel, injury, constitutional symptoms, fever, weight loss, chemotherapy with L Asparaginase or thalidomide, and so forth. A detailed dietary history for any features to suggest B12 or folic acid deficiency is absolutely essential. Look also for physical signs of B12 deficiency (like knuckle hyperpigmentation, premature graying of hair, neurological findings), signs of polycythemia (congested conjunctiva, ruddy complexion, palmar erythema), liver disease including nonalcoholic fatty liver disease (NAFLD) and features of any vasculitic disorder or any findings to suggest malignancy.

### 4.1. Diagnostic Approach

It should be individualized depending on the clinical setting.

#### 4.1.1. How to Recognize CVT?

1. Sudden onset of headache, seizure or neurologic deficit which is difficult to be attributed to one vascular territory, or unexplained vascular headache, which is persistently unilateral or sometimes diffuse.
2. CT is reported as normal/or reported as ICSOL/glioma/infarct/haemorrhage not confined to any specific vascular territory.
3. No other diagnosis/or confusion in diagnosis or reported as posterior ischemic leukoencephalopathy/demyelinating plaque.
4. Risk factors or clinical setting to develop venous thrombosis.

If all the above four are present it is almost always CVT (unless otherwise proved beyond doubt by evaluation, investigation and follow up) and hence it is wiser to be managed as CVT. Once CVT is suspected MR and MR venogram usually helps to confirm but difficult to rule out till another diagnosis is established with certainty.

The Diagnostic steps should involve the following.

1. Recognize—CVT by applying, good clinical skill and a high index of suspicion, most important step is this.
2. Rule out other possible diagnosis—by supporting investigations like CT and MRI, or sometimes only MR/MR venogram helps to rule in CVT.
3. Clinical evaluation to assess risk factors of thrombophilia by history and physical examination and supporting lab tests—even when there is no confirmed thrombus in one of the venous sinuses.
4. Identify all the possible acquired causes—investigate when necessary, if CVT is a the cause or strong possibility.
5. Look also for possible hereditary causes—if identified or strongly suspected consider prolonged anticoagulation and avoidance of all acquired risk factors for thrombosis.

One can suspect hereditary causes of thrombophilia in the following settings: absence of acquired causes, documented venous thrombo embolism in first degree relatives, early age of onset (<45 years), spontaneous and unprovoked thrombosis, recurrent episodes of venous thromboembolism, thrombus in unusual locations, thrombosis on OCP, recurrent fetal loss [1, 2, 6].

**Lab Studies**

After making the diagnosis of CVT, lab studies are necessary to look for the various contributory factors for the prothrombotic state. Complete blood count and ESR might give evidence of polycythemia (high Hb, PCV, and low ESR), B12 deficiency (low Hb, cytopenia, high MCV), very high ESR might favour a collagen vascular disorder. ANA and Anti-Ds DNA are important in suspected collagen vascular disorder. Antiphospholipid and anticardiolipin antibodies are to be done when primary or secondary APLA syndrome is suspected. Elevated SGPT and serum protein may help to screen for liver disease. Decreased albumin: globulin ratio, with hyper gammaglobulinemia can suggest hyper viscosity states. Sickle cell preparation or hemoglobin electrophoresis may be required in relevant cases. Urine protein to screen for nephrotic syndrome, D-dimer values may be beneficial in screening patients for venous thrombosis. Evaluation for protein S, C, antithrombin III, lupus anticoagulant, and factor V Leiden mutation should not be made while the patient is on anticoagulant therapy, and as such these disorders are rare as compared to several other acquired disorders which are easily managed. Lumbar puncture is helpful in evaluating for meningitis as an associated infectious process if there is clinical evidence of it. A large unilateral hemispheric lesion or posterior fossa lesion demonstrated on CT scan or MRI is a contraindication for lumbar puncture. In the past, compression of the jugular vein unilaterally with pressure measurement has been utilized which adds little to the diagnosis, and is usually not performed. EEG may be helpful in evaluating a seizure focus, may be normal, may show mild generalized slowing, or show focal abnormalities if a unilateral infarct occurs, but it does not genuinely influence diagnosis and management [1–3, 5, 6, 9, 10].

### 4.2. Mortality/Morbidity

A mortality rate of 10–80% has been reported although the higher rate is based on older data. Recent studies estimate a morbidity range of 6–20%, including residual focal neurologic deficits and blindness secondary to optic nerve atrophy. The prognosis for return of function is believed to be somewhat better than for arterial stroke. Most often the prognosis is excellent especially when the underlying disorders are promptly recognized and corrected [3, 7].

## 5. Management and Outcome

If promptly diagnosed and all the contributory factors are identified and corrected, most often it is easily managed and the patients have an excellent outcome as compared to other cerebrovascular accidents. Correct all the identifiable and reversible acquired thrombophilic states. Give intravenous fluids for dehydration, venesection in polycythemia; B12, folic acid and pyridoxine in hyperhomocysteinemia; antiplatelet drugs and drugs for reducing platelet count in extreme thrombocytosis by using Hydroxyurea, aspirin and steroid in APLA syndrome, steroid and immunosuppressants in SLE or other vasculitis disorders, antibiotic and antifungal agents in suspected infective thrombosis. Heparin is given in small doses as unfractionated heparin 2500 units to 5000 units s/c bid even when there is some haemorrhagic transformation. Higher doses can be considered if there is no haemorrhagic transformation. Dexamethasone may be used to reduce the severe intracranial pressure, unless there is a definite contraindication for using it. Mannitol can be used along with dexamethasone to reduce intracranial tension rapidly. The combination of acutely increased intracranial pressure and large venous infarcts is dangerous, and patients can die within hours from cerebral herniation. Impaired consciousness and cerebral hemorrhage are associated with a poor outcome, but in practice even patients with these manifestations can make a remarkable recovery, if treated promptly by an early clinical diagnosis. Head should be kept elevated to 30–40 degrees and supplemental oxygen may be given if level of consciousness is decreased. Seizures should be treated with appropriate anticonvulsants. The priority of treatment in the acute phase is to stabilize the patient's condition and to prevent or reverse cerebral herniation [1, 2, 5, 6, 9, 10].

### 5.1. The Issue of Anticoagulation [11–13]

The most important treatment option is anticoagulation with heparin to arrest the thrombotic process and to prevent pulmonary embolism, which may complicate venous sinus thrombosis. Anticoagulant treatment has raised much controversy because of the tendency of venous infarcts to become hemorrhagic: about 40 percent of all patients with sinus thrombosis have a hemorrhagic infarct even before anticoagulant treatment is started. The main reason to avoid heparin has been concern about its safety. But clinical trials show no increased or new cerebral hemorrhages developing after treatment with heparin. Most physicians now start treatment with heparin as soon as the diagnosis is confirmed, even in the presence of hemorrhagic infarcts. No studies have compared the effect of fractionated heparin with that of unfractionated heparin. Conventional unfractionated heparin is enough for all practical purposes. After three to four days of heparin therapy or after the patient has shown steady improvement, warfarin is started at minimally effective doses, both are overlapped for two days and then heparin is withdrawn. But the optimal duration of oral anticoagulant after the acute phase is decided by the individual patient profile. Usually oral anticoagulants are given for six months after a first episode of venous sinus thrombosis, or longer when there is persistence of the predisposing factors.

### 5.2. Thrombolysis [11]

Endovascular thrombolysis can be attempted with the administration of a thrombolytic enzyme, usually urokinase, into the sinus, sometimes in combination with mechanical thromboaspiration. Published reports are limited to case reports and uncontrolled studies, from which it is not possible to conclude that the results associated with endovascular thrombolysis are superior to those with systemic heparin. Until better evidence is available, endovascular thrombolysis may be applied at centers where the staff have experience in interventional radiology, and this treatment method should be restricted only to patients with a poor prognosis [14]. A randomized trial is probably needed to compare the effect of heparin with that of endovascular thrombolysis in selected high-risk patients. In cases of severe neurological deterioration, open thrombectomy and local thrombolytic therapy have been described as beneficial [11–13, 15, 16].

**Surgical Care**

In cases of severe neurological deterioration, open thrombectomy and local thrombolytic therapy have been described as beneficial in patients with subdural empyema or brain abscess.

### 5.3. Persistent Intracranial Hypertension

In patients who have symptoms of chronic intracranial hypertension only, the first priority is to rule out an intracranial space occupying lesion and to see whether venous sinus thrombosis could be the cause for it. If there are no contraindications, such as large infarcts or hemorrhages, a lumbar puncture is then performed to measure the cerebrospinal fluid pressure and also as a therapeutic measure. Oral acetazolamide may reduce the intracranial pressure, which may be continued for weeks to months, as is practiced in patients with idiopathic intracranial hypertension. If repeated lumbar punctures and treatment with acetazolamide do not control the intracranial pressure within about two weeks, surgical drainage of the CSF is indicated, usually by a lumboperitoneal shunt [15, 16].

## 6. Summary

More and more cases of cerebral venous thrombosis are now being diagnosed and important advances have been made in our understanding of the pathophysiology of it. Even then cerebral venous sinus thrombosis remains a diagnostic challenge and is a potentially lethal disease. High index of suspicion and awareness of the clinical features and the predisposing factors helps in diagnosis as illustrated by the two case histories below. With improved diagnosis and prompt treatment it is possible to achieve an excellent outcome for most patients. An acquired prothrombotic risk factor or a thrombophilic state can be identified in most patients with sinus thrombosis. Often, one obvious precipitating factor, such as pregnancy, delivery or postpartum state, or head injury or trauma causes sinus thrombosis in a person with one or more acquired and often subclinical thrombophilic sates or rarely with a genetically increased risk. During the last trimester of pregnancy and after delivery, the risk of venous sinus thrombosis is increased many times [3–5].

**Case History 1**

**Figure 2:**
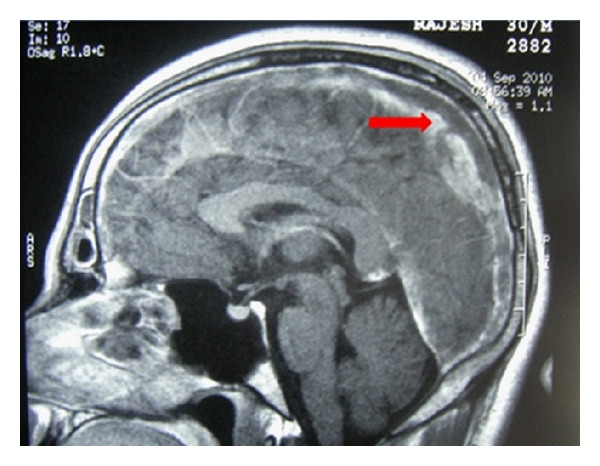
MRI scans showing cortical venous thrombosis.

Thirty year old mechanic came with sudden onset of head ache of four days duration, the headache was more on the left side. This was associated with several episodes of vomiting. No history of seizures, altered sensorium, loss of consciousness, weakness, bowel or bladder symptoms, neck pain, visual blurring. There was no head injury/trauma/ear discharge/fever, cough, breathlessness, chest pain. He was a strict vegetarian and did not consume adequate green leafy vegetables and fruits. He had left leg swelling two years back diagnosed as deep vein thrombosis. Was on Warfarin for several months which was stopped only two months back. There was no history of hypertension, Diabetes, Ischemic heart disease or similar illness in the past. He did not smoke or drink alcohol and did not have any addictions. From the history a diagnosis of cerebral vein thrombosis/sub arachnoid hemorrhage or vascular headache were considered. On examination he was conscious, oriented, cooperative, with a BMI of 22, pulse rate of 64/min, BP 140/90. No pallor/jaundice/cyanosis/clubbing/LNE/edema. Skin showed Knuckle hyper pigmentation and premature graying of hair suggesting B12 deficiency [4]. Abdomen, cardiovascular system and respiratory system were normal. Pupils were equal and reacting, No cranial nerve palsy, No motor/sensory involvement, No neck stiffness, No cerebellar signs, Skull and spine normal, Fundus-No evidence of papilloedema. On Investigations, Hb 11.6 g/dL, TLC 12000/mm^3^, DLC P 71 L 21 E 8, MCV 97.2fl, MCH 32.3, MCHC 33.2, RDW 16.1%, Platelet count was 2,78000/mm^3^, ESR 15 mm in first hour. CT head was normal, since the clinical diagnosis was CVT proceeded with MRI and MR venogram which confirmed the diagnosis of Sagittal sinus thrombosis (Figures 1 and 2). Serum homocysteine was elevated 16.61 micromol/L (normal 5–15 micromol), all other investigations including screening for collagen vascular disease, APLA, and vasculitis did not yield any positive results. The final diagnosis was cortical vein thrombosis due to hyperhomocysteinemia of Vit B12 deficiency. He was treated with Inj heparin 5000 iu iv Q6h, Inj Mannitol, Inj. vitamin B12, Inj Dexamethasone, later warfarin was added and titrated according to INR. Folic acid and multivitamin tablets were given from day one.

The case history illustrates that if CVT was not suspected by history the diagnosis could be missed, and if a detailed history of diet and lifestyle is not asked for, B12 deficiency could have been missed.

**Case History 2 [4]**

**Figure 3:**
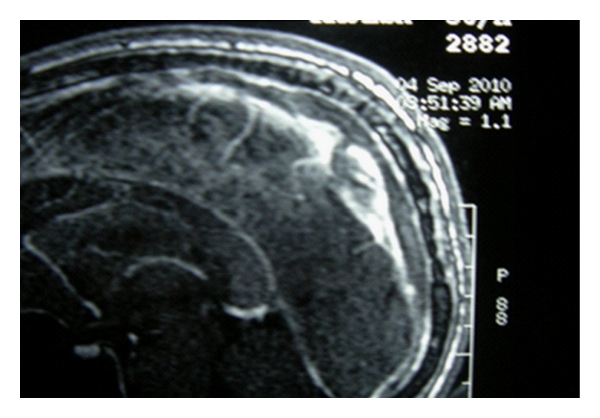
MRI scans showing cortical venous thrombosis.

**Figure 4:**
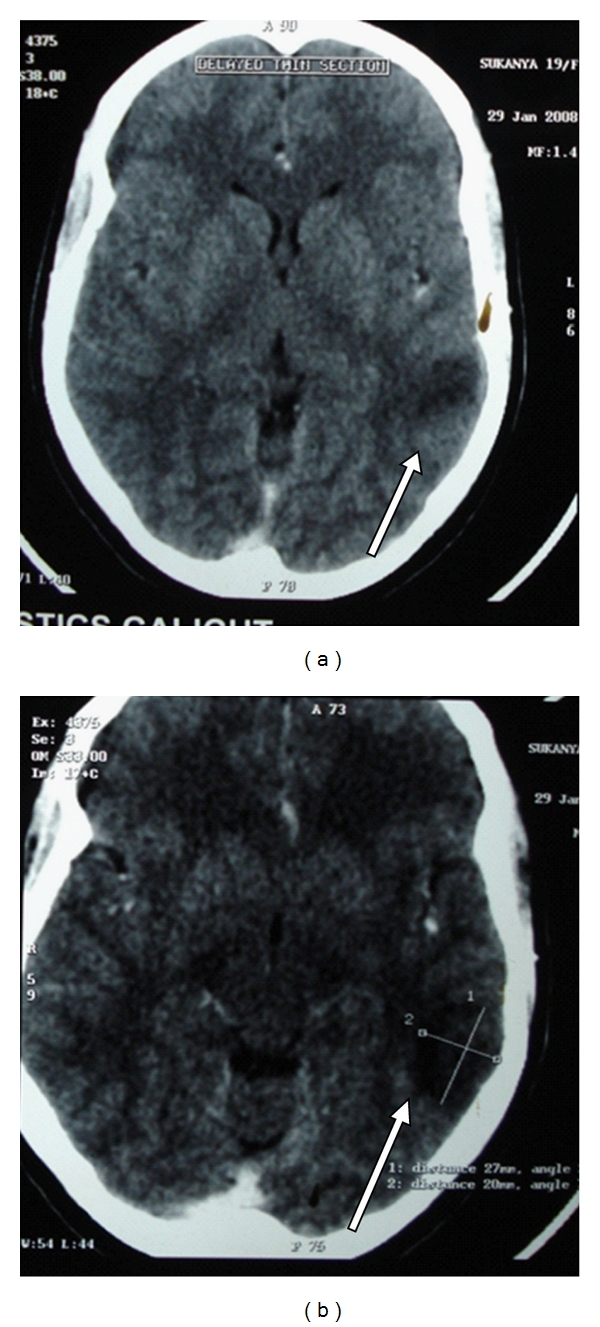
Plain CT scan showing hypodense lesion mistaken for glioma (actually venous infarct).

**Figure 5:**
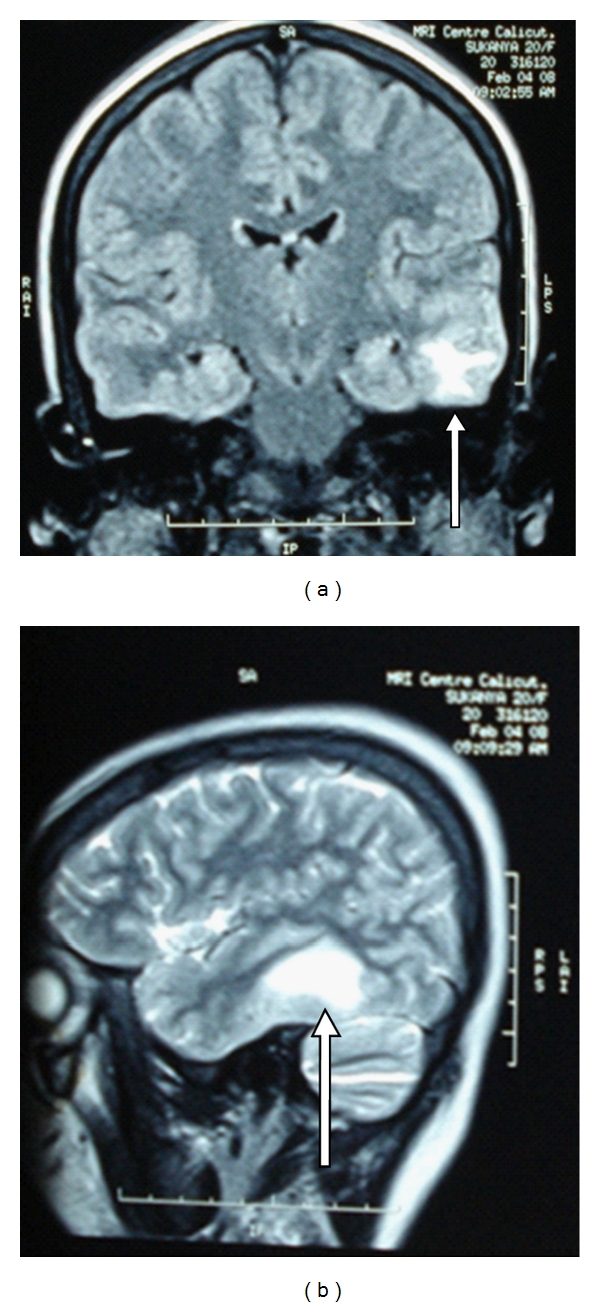
Hyperdense lesion in T2 weighted MRI scan suggesting glioma.

**Figure 6:**
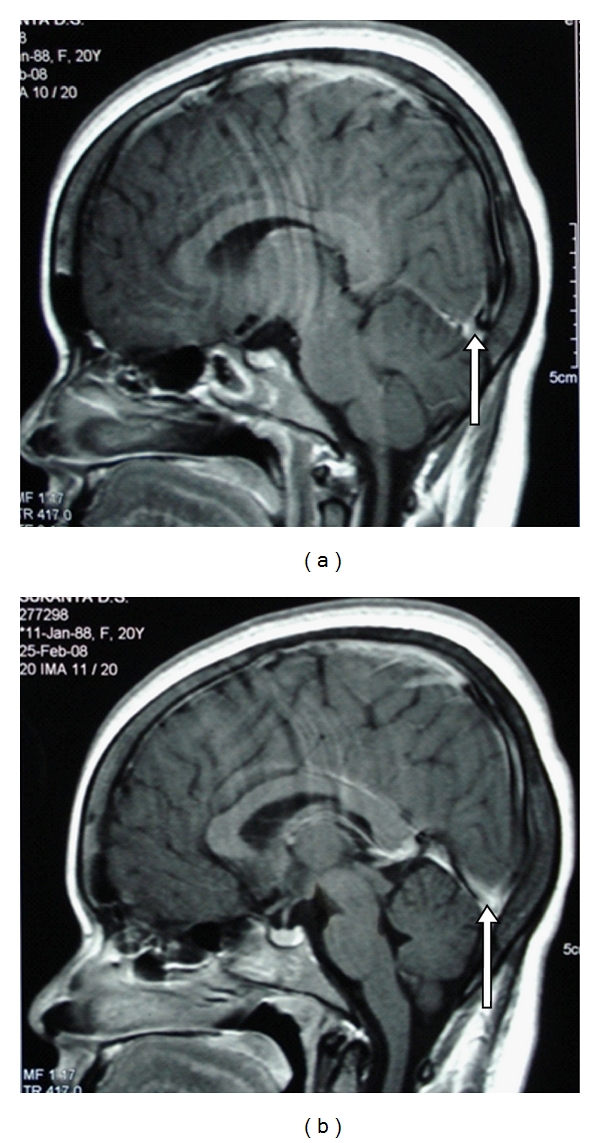
MRI scan showing hyperintensity along the course of vein of Labbe.

Twenty year old female who was asymptomatic till four months back, went to her doctor with recurrent episodes of persistently left sided headache of four months duration, subsiding with analgesics. It was not associated with any sensory or motor complaints and there was no history of early morning headache. Two months after the onset of headache she developed generalised tonic-clonic seizures, while watching television. CT head showed isodense to hypodense non-enhancing lesion with peripheral hypodense zone in the upper posterior part of the left temporal lobe and adjacent parietal lobe with minimal mass effect (Figure 3). A differential diagnosis of low grade glioma/granuloma/focal infarction/resolving hematoma was considered. MRI showed hypodense lesion in T1 weighted images and hyperdense lesion in T2 weighted images in left temporal lobe which supported the diagnosis of low grade glioma (Figures 4 and 5). Then patient was referred form the local hospital for surgery to another higher centre. Prior to surgery at the higher centre she underwent a repeat MRI which showed focal gyral hyperintensity in the region of left posterior temporal gyrus on T1 weighted images. Corresponding areas showed signal intensification on T2 weighted images. Hyperintensity was noted also along the course of vein of Labbe in F1 3D images suggesting vein of Labbe thrombosis (Figure 6). MR venogram subsequently confirmed the cortical vein thrombosis.

She was further evaluated at the higher centre, the physical examination was unremarkable according to them except for the pallor. The Hb was 9.2 gm/dL, with macrocytosis (MCV110fl), MCH 33.8 and MCHC 36, TLC and DLC was normal with mild thrombocytosis (5.1 lakh/cmm) RDW of 18%, with a normal ESR and reticulocyte count (0.6). ANA, Anti-DS DNA, Anti-phospholipid antibody were negative. The CSF study showed opening pressure of 110 mmof CSF with total of 2 cells/cmm which were lymphocytes and the protein was 41 mg/dL with sugar of 70 mg/dL (corresponding blood sugar of 99 mg/dL). The ECG and chest skiagram were normal as well as the 2D echocardiogram. Sleep EEG showed mild degree of focal nonspecific electrophysiological abnormalities over the left posterior temporal region. No epileptiform abnormalities seen. The striking abnormality was the serum homocysteine of 65 micro mol/L (normal 5–15 micromol/l). She was treated with antiepileptics and was supplemented with folate for the macrocytic anemia and for hyperhomocysteinemia. Vitamin B12 was not supplemented adequately from that centre. She was referred to us for evaluation of anemia and the pro-coagulant state.

A detailed review of history showed that she was on a strict vegetarian diet with no consumption of egg or dairy products. The vegetarian diet she took was erratic and totally unbalanced without adequate intake green leafy vegetables. She was moderately built and nourished with mild pallor, and striking premature greying of the hair and knuckle hyper-pigmentation. She had by then features suggestive of mild Subacute combined degeneration of spinal cord as evidenced by Romberg's sign positivity. Folate supplementation alone which she received prior to coming to our centre, in the presence of severe Vitamin B12 deficiency might have precipitated the neurological signs. Thus a clinical diagnosis of vitamin B12 deficiency was made. The following investigation were done: Peripheral smear showed moderate anisopoikilocytosis with normochromic normocytic cells, macroovalocytes, tear drop cells and a few microcytes suggestive of dimorphic anemia. Serum vitamin B12 was 293 pg/mL (211–911 pg/mL) and the serum ferritin was decreased. Peripheral smear report and lower limit of vitamin B12 in spite of partial supplementation earlier, supported the diagnosis of vitamin B12 deficiency with iron deficiency. Hyperhomocysteinemia was secondary to B12 deficiency which resulted in hypercoagulable state. Patient was treated with vitamin B12 and folate and she improved dramatically over the next few weeks. Currently she is asymptomatic and is adhering to balanced diet and is not on anticoagulants for the last two years. Thanks to the preoperative diagnosis of cortical venous thrombosis by the good radiologist which saved her and her family from the agony of an unwanted surgery and its complications.

This case history illustrates that if we do not elicit the dietary history and if we fail to notice the physical signs of B12 deficiency it will prolong the agony even after the diagnosis of CVT is made. It also tells that the thrombus may not be demonstrable in major sinuses at the time of evaluation. Therefore, clinical suspicion, proper clinical evaluation, supporting investigations, and clinical judgment should be the most important in arriving at the conclusions.

### 6.1. Atherothrombotic Properties of Homocysteine

Hyperhomocysteinemia can lead to vascular events like acute coronary syndromes, recurrent coronary events, stroke and venous thrombosis. It can be familial or acquired due to vitamin deficiencies. Homocysteine has primary atherogenic and prothrombotic properties. Histopathologic hallmarks of homocysteine-induced vascular injury include intimal thickening, elastic lamina disruption, smooth muscle hypertrophy, marked platelet accumulation, and the formation of platelet-enriched occlusive thrombi. Homocysteine promotes leukocyte recruitment by upregulating monocyte chemoattractant protein-1 and interleukin-8 expression and secretion. The thiolactone metabolite of homocysteine can combine with LDL-cholesterol to produce aggregates that are taken up by vascular macrophages in the arterial intima; these foam cells may then release the lipid into atherosclerotic plaques. Homocysteine increases smooth muscle cell proliferation and enhances collagen production. Prothrombotic effects of homocysteine, which have been demonstrated in patients with acute coronary syndromes include attenuation of endothelial cell tissue plasminogen activator binding sites, activation of factor VIIa and V, inhibition of protein C and heparan sulfate, increased fibrinopeptide A and prothrombin fragments 1 and 2, increased blood viscosity, and decreased endothelial antithrombotic activity due to changes in thrombomodulin function. Oxidative stress by free radicals formed during the oxidation of reduced homocysteine may directly injure endothelial cells. Marked platelet accumulation may be secondary to direct proaggregatory effects of homocysteine or to an impairment in endothelium-mediated platelet inhibition. Prolonged exposure of endothelial cells to homocysteine reduces the activity of dimethylarginine dimethylaminohydrolase, the enzyme that degrades asymmetric dimethylarginine, an endogenous inhibitor of nitric oxide synthase; this impairs the production of nitric oxide. This may contribute to impaired endothelium-dependent vasodilation of both conduit and resistance vessels [1, 4].

Vitamin B12, folate and pyridoxine deficiency contributes to development of hyperhomocysteinemia. Randomised control trials support treating all hyperhomocysteinemic patients with venous thrombosis with folic acid (1 mg/day), vitamin B6 (10 mg/day), and vitamin B12 (0.4 mg/day). All patients should receive a B complex vitamin to mitigate against peripheral neuropathy. Normalization of the homocysteine concentration has been reported within two weeks, but further lowering of homocysteine levels occurs by six weeks. Above all the importance of adherence to a balanced diet should be stressed to all patients to prevent life threatening thrombotic events. It can be postulated that folic acid and Iron deficiency with hyperhomocysteinemia and thrombocytosis in the already prothormbotic state could be the causes in many patients who develop post partum cortical vein thrombosis [4].
